# Influence of Microalgae Diets on the Biological and Growth Parameters of *Oithona nana* (Copepoda: Cyclopoida)

**DOI:** 10.3390/ani11123544

**Published:** 2021-12-14

**Authors:** Jordan I. Huanacuni, Renzo Pepe-Victoriano, María C. Lora-Vilchis, Germán E. Merino, Fressia G. Torres-Taipe, Luis A. Espinoza-Ramos

**Affiliations:** 1 Área de Biología Marina y Acuicultura, Facultad de Recursos Naturales Renovables, Universidad Arturo Prat, Avenida Santa María 2998, Arica 1000000, Chile; jhuanacunip@unjbg.edu.pe (J.I.H.); rpepev@unap.cl (R.P.-V.); 2 Programa de Magíster en Acuicultura Mención Cultivos de Recursos Hidrobiológicos Mención Acuaponía, Universidad Arturo Prat, Avenida Santa María 2998, Arica 1000000, Chile; 3 Facultad de Ciencias Agropecuarias, Escuela Profesional de Ingeniería Pesquera, Universidad Nacional Jorge Basadre Grohmann, Av. Cusco s/n, Tacna 23004, Peru; emmarie15_96@hotmail.com; 4 Centro de Investigaciones Biológicas del Noroeste, Instituto Politécnico Nacional, 195. Col. Playa de Santa Rita Sur. C. P., La Paz 23096, Baja California Sur, Mexico; cony04@cibnor.mx; 5 Departamento de Acuicultura, Facultad de Ciencias del Mar, Universidad Católica del Norte, Larrondo 1281, Coquimbo 1780000, Chile; gmerino@ucn.cl

**Keywords:** larviculture organism, live-feed production, marine copepods, phytoplankton diet, zooplankton culture, zooplankton diet

## Abstract

**Simple Summary:**

The success of marine fish farming is primarily determined by diet in early life. While both artemia and rotifers are commonly used as live feed in aquaculture laboratories in Peru, there has been little work studying the use of native food species. Here, we report our research on the use of a native copepod (*Oithona nana*) for its potential use in Peruvian marine aquaculture. We collected specimens of the native copepod *O. nana* and performed culturing experiments with two microalgae that have been widely used in aquaculture. Results show that this species adapted positively to our culture conditions, achieving high densities. We also report on the copepod’s reproduction and growth characteristics. Because of these promising results, we recommend that *O. nana* be studied further and propose it as a species with potential use as a live feed.

**Abstract:**

Several species of the planktonic free-living genus *Oithona* have been successfully used in the larviculture of marine fish and shrimp. However, few studies have been published that allow us to estimate the potential of *Oithona nana* culture under controlled conditions. This work evaluated the effect of the microalgae *Isochrysis galbana* and *Chaetoceros calcitrans* as single (200,000 cells/mL) and mixed diets (100,000 + 100,000 cells/mL) on population and individual growth, ingestion rate, number of spawnings, fertility, development time by stage, and sex ratio of *O. nana*. We cultured this copepod at 28 ± 0.5 °C, 35 PSU salinity, 125 lux, and 12:12 photoperiod. Results showed that diet had no effect on the final population level (6273–7966 ind/L) or on individual growth, nor on sex ratio, with less males than females. With *C. calcitrans*, *O. nana* had a higher filtration rate (57 ng C/ind/day). On the other hand, a mixed diet induced a higher number of spawns (0.4 events/day) and nauplii per spawn (23 ind). Similarly, a single or mixed diet, containing *I. galbana,* accelerated the development rate by 6.33–7.00 days. We concluded that *O. nana* can be cultured with both microalgae, indicating its potential use in an intensive system for production. However, more research is required to improve the productivity of *O. nana* rearing.

## 1. Introduction

Zooplanktonic copepods form part of the diet of many aquatic organisms [1], and because of this they have been employed as live prey in fish larviculture [2]. Copepods are a good food source for fish larvae because they have a better biochemical profile than traditional live feeds such as rotifers and *Artemia* [3,4]. Researchers have shown that copepods increase fish larvae survival, growth, health, pigmentation, and tolerance to the stressors of rearing conditions [5,6,7,8,9].

Since the 1960s, approximately 60 copepod species have been reared as live-feed [10]. Among the most frequently studied are the calanoids *Acartia* spp. [11,12,13], *Calanus* spp. [14,15,16], the cyclopoids *Oithona* spp. [17,18,19], *Apocyclops* spp. [2,20,21], the harpacticoids *Tisbe* spp. [22,23,24], *Tigriopus* spp. [25,26,27]. Despite the progress made, many species of copepods that present aquaculture potential remain unstudied [28]. This is particularly true for cyclopoid copepods, which are as common as calanoids in marine systems.

Having been reported in various parts of the world [29,30,31,32,33], the genus *Oithona* [34] has possibly the greatest distribution and abundance in the marine environment [35]. In addition, they are considered to provide an important link between primary producers and fish larvae [36] and are recognized as a part of the diet of commercially significant marine species [3,37]. As a result, some species of *Oithona* are used as live feed in marine aquaculture and others are considered candidates [38]. Immunostimulants, attractants, and some digestive enzymes are found in this genus [3,37].

The species *Oithona nana* [39] is part of the most dominant planktonic copepods in the world’s oceans [40,41]. It is found in neritic and oceanic zones [29,30,31,32,33,42,43], with a preference for warm waters [44]. According to Berraho et al. [29], Berraho et al. [30], La-vaniegos et al. [45], and Magouz et al. [46], *O. nana* has a very varied diet and a low metabolic rate, all of which are advantageous qualities for rearing purposes. According to Lampitt and Gable [47], *O. nana* is euryphagic, with a varied diet. It is capable of consuming phytoplankton, bacteria, protozoa, and small crustaceans [47,48]. It can also consume yeast and flour from vegetable sources [7,46,49,50].

The use of native species as live feed in aquacultural has previously been proposed [51], but reports on the practical use of *O. nana*, a species prevalent along the Peruvian coast, are scarce. The objective of our research was to obtain information on the cultivation of *O. nana.* To do so, we studied its developmental stages and evaluated its growth and biological parameters when fed two microalgae diets commonly used in conventional aquaculture laboratories [52,53,54]. These studies were carried out to establish its potential as an alternative source of live feed for marine fish larviculture.

We hypothesize that the different stages of *O. nana* can be cultured in controlled conditions using either monospecific or mixed diets of the microalgae *Isochrysis galbana* and *Chaetoceros calcitrans*.

## 2. Materials and Methods

### 2.1. Maintenance of Microalgae and Copepods

The microalgae cultures *I. galbana* and *C. calcitrans* (Table 1) were obtained from the Centro de Acuicultura de Morro Sama (CAMOSA) Live Feed Laboratory, which is located at 18°00′04.6″ S 70°53′11.9″ W, Tacna, Peru. The microalgae were grown in medium f/2 [55] at 23 ± 2 °C, 35 PSU salinity, and a photoperiod of 24 h light. The seawater used in this study was filtered at 0.22 μm, UV-treated, and autoclaved (1 atm, 121 °C).

The copepod *O. nana* was captured in the summer of 2019 from the rocky beaches of CAMOSA, using a 55 μm plankton collector, and they were transported to the live feed stock of the Universidad Nacional Jorge Basadre Grohmann (UNJBG), which is also located in CAMOSA. The copepods were identified by the Ornithology and Biodiversity Center (CORBIDI) in Lima, in accordance with Murphy [48] and Ferrari and Bowman [59]. The copepods were kept in 1 L flasks at 28 ± 0.5 °C, 35 PSU salinity, 125 lux, and a 12:12 photoperiod, at a density of less than 5 ind/mL, with a low level of constant aeration. We fed the copepod cultures a mixed diet of *I. galbana* and *C. calcitrans* at a density of 10^6^ cells/mL of each species, with 100% water exchange every 15 days. Seawater was filtered through a 0.45 μm nitrocellulose membrane using a vacuum pump (Vacuubrand MD-1, Wertheim, Germany) and then treated with UV (American Ultraviolet AL-PVC-160W, Lebanon, IN 46052, USA).

### 2.2. Experimental Design

For each experiment, the copepods were fed with only one of the three experimental diets at a density of 200,000 cells/mL. The experimental diets were: *Iso*, *I. galbana* (2400 μg C/L); *Ch*, *C. calcitrans* (3100 μg C/L); and *Iso* + *Ch*, *I. galbana* plus *C. calcitrans* (1200 + 1550 μg C/L). Assays were used to determine the required concentration of microalgae before conducting the experiments. All cultures were obtained at 28 ± 0.5 °C, 35 PSU salinity, 125 lux, and a 12:12 photoperiod, with a slight and constant aeration in UV-treated filtered (0.45 μm) seawater. The algal concentration was measured daily in all cultures using a Neubauer chamber (mean of six-cell counts), and corresponding volumes of micro-algae were administered to maintain stability in the experimental concentration.

### 2.3. Population and Individual Growth and Ingestion Rate

To begin with, 10 sexually mature pairs of *O. nana* were placed into five 1-L flasks, each containing 800 mL of filtered seawater. The experimental conditions were kept constant for 16 days, with manual shaking every 12 h from the third day onwards. Finally, the population of each experimental unit was harvested using a 50 μm sieve and preserved in a 3.7% formalin solution of 10 mL to be counted later by stage (nauplii, copepodites, adults: male and female) in a Sedgwick-Rafter chamber. The total length (prosome + urosome) of 600 individuals, collected from each flask (150 per stage), was measured with a microscope (Optika B-500Ti, OPTIKA S.r.l., Ponteranica, BG, Italy) equipped with a previously graduated micrometer eyepiece. Microalgae consumption was measured in 240 adults of *O. nana* between 24 to 48 h in the 1 L flasks, under the same conditions and at the same time as the growth experiment. This data was compared to the growth of single algae cultures, to assess the actual ingestion of microalgae. The ingestion rates (I, cells/mL h) were determined according to equation, indicated by Frost [60]:

I = (C1 − C2)/(N × 24),

where, C1 and C2 are the concentrations of microalgae (cells/mL), N is the number of copepods, for a duration of 24 h.

### 2.4. Number of Spawning and Fertility

Females who had reached their first sexual maturation (i.e., possessing ovigerous sacs) were isolated from a stock culture and placed in a 100 × 20 mm Petri dish filled with 40 mL of media (one individual per dish), with single or mixed algal food at the experimental density, and sealed with parafilm. The experiment was carried out in triplicate. When the females spawned, the nauplii that were released were quickly collected and placed on a new plate with a new medium. The number of spawning events for each female was recorded over the course of 20 days. We counted the nauplii for each spawn in each treatment to determine female fertility (number of nauplii per spawning).

### 2.5. Development Time by Stage and Sex Ratio

One egg-bearing female was removed from a stock culture and placed in a Petri dish with 40 mL of culture media, with single or mixed algal food at the experimental density. The experiment was carried out in triplicate. The female was examined twice a day to determine when the sac ruptured and the nauplii were released, and at this point, the female was then transferred to another Petri dish with the culture medium, leaving the nauplii alone. The development time by stage was determined by examining the Petri dishes containing nauplii every 12 h for 20 days under a stereo microscope, differentiating the molts until they reached adulthood, when sexual dimorphism was recognizable. The sex ratio (male/female) was calculated based on the number of adult copepods.

### 2.6. Analysis of Data

The normal distribution of the data was determined using the Shapiro–Wilk test, and the variance homogeneity was determined using the Levene test. The *t*-test for independent samples was used for the single algal ingestion rate and a one-way ANOVA was used to obtain the other data. A Tukey HSD test was used for results that were found to be significant (*p* < 0.05). When the data did not meet the statistical assumptions of ANOVA, the Kruskal-Wallis test was applied, and non-parametric multiple comparisons of the p-values were performed for significant results (*p* < 0.05) [61]. Statistical analyses were performed with STATISTICA 10.0 (StatSoft, Tulsa, OK, USA).

## 3. Results

### 3.1. Population and Individual Growth and Ingestion Rate

**Figure 1:**
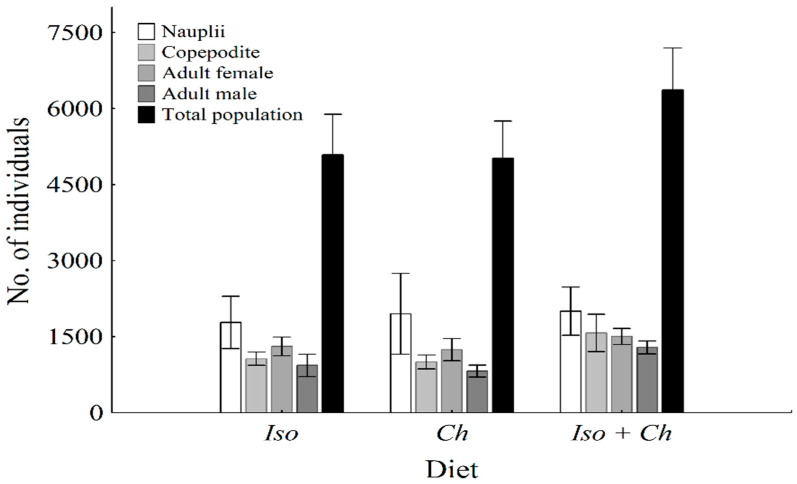
Total population after 16 days in a culture (individuals in 800 mL) of *Oithona nana* within the same stage between treatments. There was no difference found among the experimental groups. Descriptive errors bars, shown as average ± Standard Deviation (*n* = 5).

The population density on day 16 did not differ significantly between treatments (Figure 1), nor did the densities in the percentage of nauplii, copepodites, adult female, and male stages (Table 2). Table 3 and Table 4 show the length values of *O. nana* in the minimum-maximum intervals for each stage fed with different diets. Furthermore, there was no difference in the average length between the experimental groups. The ingestion rates, measured over 24 h, revealed no differences between single algae diets (Table 3).

### 3.2. Number of Spawning and Fertility

**Figure 2:**
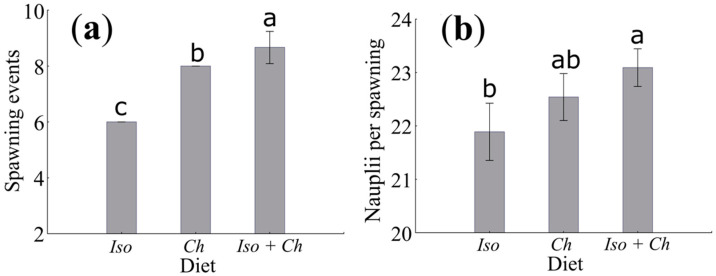
*Oithona nana* fed with single and mixed diets during 20 days of evaluation. (**a**) Number of spawnings. (**b**) Fertility or number of nauplii per spawning. Descriptive error bars shown as average ± Standard Deviation. The letters above each bar indicate the significant differences (a > b > c, *n* = 3, *p* < 0.05).

The number of spawnings of *O. nana* during a 20-day period differed significantly depending on the diet supplied (*p* < 0.05). The single *I. galbana* diet resulted in the lowest number of spawnings. Similarly, the number of nauplii per spawning showed the lowest values for the *I. galbana* diet (*p* < 0.05) (Figure 2).

### 3.3. Development Time by Stage and Sex Ratio

The development time per stage of *O. nana* during the 20-day period showed a significant difference in all stages (*p* < 0.05). In the nauplii phase, the *Iso* diet provided the shortest time (*p* < 0.05), while in the copepod phase (*p* < 0.05), the *Iso* and mixed diets provided a shorter development time, and thus a shorter time to obtain adult copepods. At the end of the experiment, the proportion of sex did not differ between treatments (*p* < 0.05) (Table 5).

## 4. Discussion

Our study provided information on the growth parameters of *O. nana* under specific, constant conditions as recommended by Kaviyarasan et al. [52] and Cornwell et al. [70]. As a result, we have a better understanding of how this species may be successfully cultured.

### 4.1. Population Growth

An evaluation of how diet effects the growth of copepod populations provides information for their potential use in aquaculture [71,72]. This was confirmed for *O. nana* using the oceanic algae *Nannochloropsis* in experiments at 2 × 10^6^ cells/mL and 5 × 10^6^ cells/mL [46]. Similar results were reported for *O. rigida* [73]. In our study, single and mixed diets with *I. galbana* and *C. calcitrans* did not affect the final population of *O. nana*. This could indicate the easy adaptation of this copepod to these algae. Chilmawati and Suminto [3] reported results similar to ours; they reported maximum population densities of 6.96 ± 0.38 ind/mL with *C. calcitrans* (2 × 10^6^ cells/mL) and 6.28 ± 0.25 ind/mL with *I. galbana* (2 × 10^6^ cells/mL) in *Oithona* sp.

In our study, we did not find significant differences among experimental algae diets, meaning that *O. nana* can be cultivated with single and mixed diets. In addition, the use of a single diet means less algae rearing hatchery work and diminishes possibility of cross-contamination in copepod cultures vessels. According to Santhanam et al. [24], *I. galbana* (30,000 cels/mL) alone is sufficient in the diet for *O. rigida* due to the high content of DHA, and the high DHA:EPA ratio required for its proper growth. The positive effect of *Iso* and *Ch* as single diets with a high content of DHA and EPA, respectively, could be attributed to the synthesis and bioaccumulation capacity of highly unsaturated fatty acids (HUFA) in copepods [8,74,75,76]. This biotransformation of essential fatty acids has been demonstrated for cyclopoid copepods [77,78], calanoid copepods [75,79], and harpacticoid copepods [74,80].

Because organisms’ behavior and growth can vary even within the same taxonomic group, a mixed algal diet may be preferable for rearing copepods such as *O. nana*, which has six naupliar phases and five copepodite stages [81]. However, because the aim of our study was to measure copepod production and the growth time between stages as a function of food, we did not characterize each stage by each growth stage.

In terms of the overall population, the final density of copepods obtained in our experiments (5.25–6.61 ind/mL) is lower than that indicated by Magouz et al. [46], who reported concentrations of more than 9 ind/mL, grown on an alternative cornstarch diet. This difference in densities of the final population could be attributed to the initial inoculum density, which included 10 adult female *O. nana* at their first sexual maturity per 800 mL in this study, as opposed to Horne et al. [81] and Magouz et al. [46], who started with 1875 nauplii/L and 1000 ind/L, respectively. Oftentimes, live feed laboratories start their mass cultures with high concentrations because it is sufficient for harvesting higher densities. However, to avoid overcrowding and negative outcomes, the maximum rearing densities must be determined for this species. High rearing densities are known to have a negative impact on growth, behavior [82], fertilization [83], adult cannibalism of nauplii [2,84,85,86,87], female reproductive capacity, egg hatching rate, and to induce the collapse of the reared stocks [84], causing the nauplii population to decline. Holm et al. [88] reported that maintaining a high density of *O. nana* males minimizes mate-seeking behavior after a period of competition for food and starvation, which also may limit the growth of the species [89]. To avoid this behavior, it would be necessary to first conduct high stoking density experiments with *O. nana* to determine whether a problem exists in the mass culture.

### 4.2. Individual Growth

Diet plays an important role in determining copepod size [90]. Our data showed no difference in body size between *O. nana* that were fed single and mixed algae diets. This is not the case in other cyclopoids, such as *Paracyclopina nana*, which were found to have larger body sizes when fed mixed diets [91]. The body size range for each stage of *O. nana* recorded in this study corresponds to the sizes reported in natural and cultured conditions by other authors, indicated in Table 4.

### 4.3. Ingestion Rate

The ingestion rate is an important metric through which to determining copepod food preferences in both their environment and under laboratory conditions [92,93]. Even though *C. calcitrans* had larger dimensions and a larger biovolume than *I. galbana* (Table 1), adults of *O. nana* did not show a difference in ingestion rate in our study. This could indicate a preference for specific nutrients found in both microalgae. However, because developing *O. nana* nauplii may have difficulty consuming and digesting the diatom *C. calcitrans* [3]—perhaps due to its long setae—they were not considered in this evaluation.

Species of *Oithona* are classified as diet-mixed consumers; adults can be carnivorous [94]. Carnivory was confirmed for adults of *O. nana* when supplied with *Acartia clausi* nauplii [95]. According to Lampitt and Gamble [47], *O. nana* can consume phytoplankton or nauplii in monospecific diets in the range of 1–300 um, as well as non-traditional foods such as soybeans, yeast, rice bran, and corn starch [46]. Thus, *O. nana* possess a high adaptability to consume a wide variety of foods.

According to our findings (Table 3), adults of *O. nana* consumed high amounts of microalgae, as evidenced by the carbon consumption of 34.07 ng C/ind/day for *I. galbana* and of 56.99 ng C/ind/day for *C. calcitrans*. These values are higher than those reported by Lampitt and Gamble [47], who evaluated the diet of the following microalgae, ranging in size from 2.7–10 μm to 10–67 μm: *I. galbana* and *Dunaliella euchlora*, *Cricosphaera elongata*, *Thalassiosira fluviatilis*, and *Prorocentrurn micans* reported a consumption of 9.2 ng C/ind/day with *I. galbana* and the highest consumption of 51.5 ng C/ind/day with *Cricosphaera elongata*, at an ambient temperature of 10 °C. In our case, we used a higher temperature (28 ± 1 °C), which could have resulted in a greater rate of ingestion of smaller microalgae. Another factor that may lead to the reduced ingestion of tiny cells is hydrodynamic disruption, which has been found in the culture of other species to alter swimming and food perception [87].

### 4.4. Number of Spawning

The development rate, longevity, and fertility of copepod are all influenced by the type and availability of food [96,97]. In our experiment, single and mixed diets containing *Chaetoceros* sp. produced greater spawning, perhaps due to the high concentration of polyunsaturated fatty acids [98]. According to Lora-Vilchis et al. [99], unlike *I. galbana*, *Chaetoceros* sp. has a high concentration of HUFAs and approximately five times the content of EPA (Table 1). However, to achieve optimal fertility, the 2:1 DHA/EPA ratio must be considered in copepod diets [96].

In our cultures, *O. nana* spawned at a high frequency during periods of 1 to 4 days when cultivated at 28 °C, with longer periods as the females’ ages increased. Haq [68] reported that embryonic phase of *O. nana* in cultures with *Phaeodactylum tricornutum* was 4 to 5 days at 18 °C and 2.5 to 3.5 days at 20 °C, and Temperoni [100] reported times of 3.22, 2.37, and 2.05 days for this species in oceanographic sampling at 12, 17 and 21 °C, respectively. Hence, we found that temperature influences embryonic and reproductive development in *Oithona* [36]. Given these data, it would be appropriate to cultivate *O. nana* at temperatures that allow it to adapt to local hatchery conditions, ideally at 28 °C, which corresponds to the temperature detected during *O. nana* collection on Morro Sama’s rocky beaches.

The high temperature of 28 °C may have contributed to the fact that *O. nana* was the only copepod species found at the sampling site, which allowed us to obtain a monospecific sample. In addition to being an organism found worldwide, *O. nana* can also be categorized as a eurytherm, as it has been found in South America at temperatures ranging from 18.5 to 37 °C [33].

### 4.5. Fecundity

According to Støttrup and Jensen [101], the size, quantity and quality of food, have a direct influence on the number of copepod eggs produced per female. Our results show that the mixed diet produced the most nauplii of *O. nana* per egg laying event. On the contrary, Chilmawati and Suminto [3] found no differences in egg production in *Oithona* sp. utilizing four single algae diets: 1–2 × 10^6^ cells/mL for *C. calcitrans* and *I. galbana*, and 2–4 × 10^6^ cells/mL for *Chlorella vulgaris* and *Nannochloropsis oculata*. This could be because different algal diets produce different reproductive responses in copepods [102]. Suminto et al. [103] successfully tested the inclusion of organic ferments in single diets with *C. calcitrans* (2 × 10^6^ cells/mL) in *Oithona* sp. to enhance egg production, and thus the number of nauplii produced. Similarly, Chilmawati et al. [104] obtained a higher level of egg production in *O. similis* fed with an organic feed containing 30% protein.

The number of nauplii per liter produced at 28 °C in our cultures was greater than that reported by Haq [68], who used the diatom *Phaeodactylum tricornutum*. Haq found 21, 22, 15, and 6 nauplii/L of cultured *O. nana* at 11, 18, 18 and 20 °C, respectively. This demonstrates that nutrition and rearing temperature affect both the number of nauplii per liter and the number of spawning events. However, as shown for the herbivorous copepod, *P. crassirostris*, eggs can be damaged or eaten by *O. nana* [47], which might cause issues in nauplii production when the density of adults increases [105].

### 4.6. Development Time Per Stage

Food quality clearly affects growth of copepods [92]. Our results indicate a longer copepod development period when they are fed with a monospecific diet of *C. calcitrans*. The nauplii and early stages of copepodites were unable to digest this diatom due to their diameter and cell wall composition. On the other hand, *I. galbana* microalgae does not have rigid cell wall, which, coupled with its smaller size may be important for food ingestion and digestion by nauplii. Of course, as the organisms grow, they may consume larger cells, thereby providing them with more energy. We found that nauplii required 4 to 7 days to grow, whereas copepodite required 2.33 to 4.67 days. Conversely, Murphy [48] demonstrated the requirement of 26 days for growth in the naupliar stage and 28 days for the copepod stage of *O. nana* fed with fresh and decomposed algae diets with the addition of *Navicula*. This would mean that diets that do not satisfy the nutritional requirements of the copepod could lead to a prolonged development time in copepods. Due to the identification of non-optimum temperatures, the results must be corroborated by additional studies, i.e., by determining the temperature coefficient (Q10) and thermal dependence. For example, Almeda et al. [106] report a Q10 of 2.4 for *O. davisae* nauplii cultured between 12 and 28 °C. Species in the genus *Oithona* have a short development time, for instance, *Oithona rigida* cultured at 28 °C presented development times from 3.8 to 5.2 days for nauplii and 7.1 to 8.4 days for copepodites, both fed with *I. galbana* at 40 × 10^3^ cells/mL [24].

### 4.7. Sex Ratio

Various studies report that the sex ratios obtained in zooplankton are not of a ratio of 1:1 [107]. Although there is a propensity for a higher percentage of females than males in *O. nana*, little variation was observed in the sex ratio in our experimental treatments (Figure 2 and Table 5). Temperoni [100] found that females outnumbered males in more than 90% of his oceanographic samplings, with females outnumbering males by 60 to 100% of the total population of *O. nana*. This also coincides with the results reported by Holm et al. [88], who claim that the male to female ratio is even lower than 20% under growing conditions. Temperature [81] and salinity [44,108,109] are two potential environmental factors that may influence the development of *O. nana,* and manifest in the predominance of one of the sexes. Another possibility that has been reported is the change of sex in planktonic copepods during their development under adverse conditions [107], which is primarily related to quantity [110] and food quality [111]. Although there have been no reports of a change in sex for *O. nana*, we recommend additional research aimed at increasing fecundity and the proportion of females. In this way, the cultivation of *O. nana* can increase in output and productivity.

Although Murphy [48] and Haq [68] were the first to study *O. nana* in culture, their objectives were to develop an understanding of the morphological description of the life cycle and the nauplii stage, respectively. Following their study, only a few studies used biological parameters to assess the viability of their cultures. Our findings are significant because they demonstrate the possibility of culturing *O. nana* with two microalgae species that are often used in aquaculture production facilities. They are also proof of the metabolic adaptability of *O. nana* in obtaining and assimilating essential nutrients from different diets to perform its complete biological cycle. This could indicate a bioaccumulation possible bioconversion of essential nutrients at various stages of development, which is important in the transfer of these compounds to higher levels of the food web. These factors have been reported on for other groups of copepods, but have yet to be investigated for *O. nana*.

## 5. Conclusions

Our study provides valuable information about culturing the copepod *O. nana* in small-scale settings. We found that *I. galbana* and *C. calcitrans* are suitable food sources across all development stages of *O. nana*. This constitutes an advantage for producing live feed for copepods that cultivate both species of microalgae as food for zooplankton. The next logical steps are to (1) assess growth while varying conditions of temperature, food quality and quantity, and water chemistry and (2) scale the culture.

## Figures and Tables

**Table 1:** *Isochrysis galbana* and *Chaetoceros calcitrans* algae general information.

	Size (µm) ^1^	Biovolume (µm^3^⋅cell^–1^) ^1^	Carbon Content (pg C⋅cell^–1^) ^1^	% ARA ^2^	% EPA ^2^	% DHA ^2^
*I. galbana*	5.7	99.5	12.0	0.11	0.32	19.55
*C. calcitrans*	7.0 × 4.9	136.2	15.4	3.86	27.06	2.75

The transformation suggested by Menden-Deuer and Lessard [56] for converting cell volume to carbon concentration was employed, which consists of using the equation 1: Log (C) = –0.541 + 0.811 (Log V), where, C is the cellular carbon concentration (pg C cell^–1^), and V is the cell volume (μm^3^). ARA: arachidonic acid, EPA: eicosapentaenoic acid, DHA: docosahexanoic acid. ^1^ [57]. ^2^ [58].

**Table 2:** Population growth of *Oithona nana* fed with single and mixed diets after 16 days in culture. The percentage of the population as nauplii, copepodites, and females and males are recorded, as well as the total population size (ind./L) by day 16. Values shown as average ± standard.

			Single Diet		Mixed Diet
			*Iso*	*Ch*	*Iso + Ch*
Production per stage (%)	Nauplii		35 ± 5	38 ± 10	31 ± 4
	Copepod		21 ± 3	20 ± 2	25 ± 4
	Adults	Female	26 ± 4	25 ± 6	24 ± 4
		Male	18 ± 4	17 ± 3	20 ± 3
Total production (individual/L)			6360 ± 998	6273 ± 919	7966 ± 1032

Standard deviation. There was no difference among experimental groups. (*n* = 5, *p* < 0.05).

**Table 3:** *Oithona nana* individual growth (μm) of nauplii, copepodites, and females and males after 16 days in culture, and ingestion rate (I) of adults after 24 h in culture fed with single and mixed diets.

			Single Diet						Mixed Diet		
			*Iso*			*Ch*			*Iso + Ch*		
			Min	Max	Average	Min	Max	Average	Min	Max	Average
(μm)	Nauplii		96.3	298.7	190.8 ± 46.6	82.4	288.4	167.6 ± 49.1	96.6	288.4	195.0 ± 53.7
	Copepodite		309.0	494.4	401.8 ± 42.8	247.2	545.9	403.3 ± 62.4	245.2	544.0	380.0 ± 58.3
	Adults	Female	535.6	690.1	620.8 ± 33.9	515.0	690.1	621.3 ± 35.9	515.0	698.3	622.4 ± 36.7
		Male	506.7	700.6	613.0 ± 60.6	535.6	690.1	611.5 ± 39.7	506.6	699.9	615.5 ± 61.2
I	cells/ind/h		118.3 ± 61.9 ^b^			154.2 ± 30.2 ^a^			—		
	ng C/ind/day		34.1			57.0			—		

Values (average ± standard deviation). Letters indicate significant differences (a > b, *N* = 150 per each development stage, *n* = 5, *p* < 0.05).

**Table 4:** *Oithona nana* length comparison (µm) fed with experimental microalgae or natural food, in the different development stages, nauplii, copepodites, and females and males.

Microalgae (×10^3^/mL)	Nauplii	Copepodite	Female	Male	Source
*I. galbana* (200) and *C. calcitrans* (200)	82.4–298.7	247.2–587.1	432.6–772.5	515.0–762.2	Present research
*Oxhyrris marina* (1) + *Rhodomonas salina* (1)			352 ± 23 **		[62]
N.E. Colombia	-		<1000	<1000	[63]
N.E. Tunez			480–800	480–800	[64]
N.E. Argentina		C-I: 276 ± 18 C-II: 327 ± 17 C-III: 382 ± 25 C-IV: 448 ± 24 C-V: 531 ± 36	609 ± 36	608 ± 49	[65]
N.E. Italy			569 ± 33	539 ± 24	[66]
N.E. Central and South America			580–720	470–530	[59]
N.E. Mediterranean sea			400–640	400–610	[67]
*Phaeodactylum tricornutum* *	N-I: 50–85 N-II: 85–100 N-III: 100–110 N-IV: 110–125 N-V: 130–145 N-VI: 150–175				[68]
N.E. Adriatic sea			520	520	[69]
Fresh and decomposed algae + *Navicula*	N-I: 40 N-II: 75 N-III: 90–97 N-IV:110–120 N-V: 130–150 N-VI: 150–190	C-I: 190–200 C-II: 260–320 C-III: 340–380 C-IV: 400–480 C-V: 450–520	480	550	[48]

* Not defined as female or male; ** Prosoma length; N.E.: Natural environment

**Table 5:** *Oithona nana* development time by stage (days) as nauplii, copepodites and adults fed with single and mixed diets during 20 days of evaluation, and sex ratio of adults.

		Single Diet		Mixed Diet
		*Iso*	*Ch*	*Iso + Ch*
Development	Nauplii	4.00 ± 0.00 ^b^	7.00 ± 0.00 ^a^	5.00 ± 0.00 ^ab^
	Copepodite	2.33 ± 0.00 ^b^	4.67 ± 0.58 ^a^	2.00 ± 0.00 ^b^
	Adults	13.67 ± 0.58 ^a^	8.33 ± 0.58 ^b^	13.67 ± 0.00 ^a^
Sex ratio	(No. male/No. female) (%Male:%Female)	0.67 ± 0.10 (38:62)	0.81 ± 0.24 (44:56)	0.75 ± 0.22 (42:58)

Values (Average ± Standard Deviation). Letters indicate significant difference (a > b, *n* = 3, *p* < 0.05)

## Data Availability

The data presented in this study are available on request from the corresponding author. The data are not publicly available for privacy reasons.
